# Acquired ductopenia: an insight into imaging findings

**DOI:** 10.1007/s00261-024-04462-x

**Published:** 2024-07-02

**Authors:** Rachita Khot, Nathan R. Shelman, Daniel R. Ludwig, Rashmi T. Nair, Mark A. Anderson, Sudhakar K. Venkatesh, Raj Mohan Paspulati, Rex A. Parker, Christine O. Menias

**Affiliations:** 1 Radiology and Medical Imaging, University of Virginia, Charlottesville, VA USA; 2 Department of Pathology, University of Kentucky, Lexington, KY USA; 3 Mallinckrodt Institute of Radiology, Washington University School of Medicine, Saint Louis, MO USA; 4 Department of Radiology, University of Kentucky, Lexington, KY USA; 5 Department of Radiology, Massachusetts General Hospital, Harvard Medical School, Boston, MA USA; 6 Division of Abdominal Imaging, Department of Radiology, Mayo Clinic, Rochester, MN USA; 7 Department of Diagnostic Imaging and Interventional Radiology, Moffitt Cancer Center, Tampa, FL USA; 8 University of Kansas Medical Center, Kansas City, KS USA; 9 Division of Abdominal Imaging, Department of Radiology, Mayo Clinic, Scottsdale, AZ USA

**Keywords:** Ductopenia, Vanishing bile duct syndrome, Autoimmune diseases, Ischemic cholangitis, Drug-induced injury, Graft-versus-host disease

## Abstract

Hepatic ductopenia is a pathologic diagnosis characterized by a decrease in the number of intrahepatic bile ducts as a consequence of various underlying etiologies. Some etiologies, such as primary sclerosing cholangitis, primary biliary cholangitis, and ischemic cholangitis, often have distinctive imaging findings. In contrast, other causes such as chronic rejection following liver transplantation, drug-induced biliary injury, infection, malignancy such as lymphoma, and graft-versus-host disease may only have ancillary or non-specific imaging findings. Thus, diagnosing ductopenia in conditions with nonspecific imaging findings requires a multidimensional approach, including clinical evaluation, serological testing, imaging, and liver histology to identify the underlying cause. These etiologies lead to impaired bile flow, resulting in cholestasis, liver dysfunction, and, ultimately, cirrhosis and liver failure if the underlying cause remains untreated or undetected. In the majority of instances, individuals diagnosed with ductopenia exhibit a positive response to treatment addressing the root cause or cessation of the causative agent. This article focuses on acquired causes of ductopenia, its clinical manifestation, histopathology, imaging diagnosis, and management.

**Graphical abstract:**

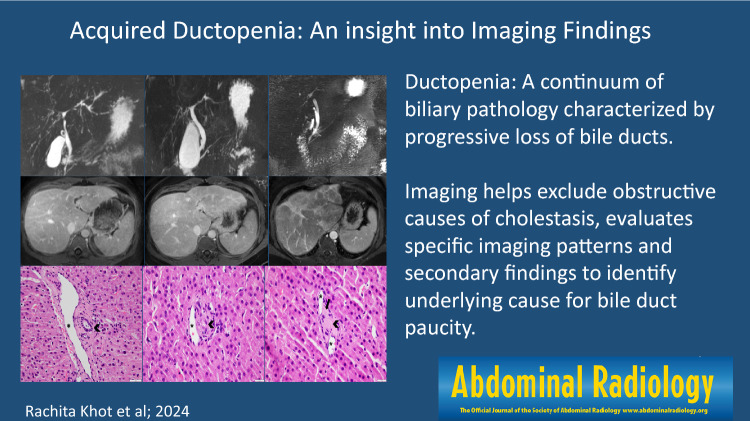

## Introduction

Ductopenia is a semiquantitative term characterized by a pathologic reduction of intrahepatic bile ducts with resultant cholestasis. Vanishing bile duct syndrome (VBDS) is a rare and severe form of ductopenia characterized by the progressive loss or complete disappearance of intrahepatic bile ducts [1]. Ductopenia is associated with congenital and acquired etiologies. This article focuses on acquired etiologies including autoimmune diseases, ischemic cholangitis, drug-induced liver injury, infections, malignancy, and graft-versus-host disease. The hallmark feature of ductopenia is chronic cholestasis, serum alkaline phosphatase ≥ 2 times the upper limit of normal, and the absence of interlobular bile ducts in more than 50% of small portal tracts in an adequate biopsy sample [2]. The pathogenesis of ductopenia involves a complex interplay of immune-mediated mechanisms and hepatobiliary injury, which have not been fully elucidated. Diagnosis requires a comprehensive evaluation, including clinical presentation, laboratory investigations, imaging, and liver histology. Management primarily focuses on treating the underlying cause, if identified, and providing supportive care to manage complications such as pruritus. In severe cases, liver transplantation or re-transplantation may be necessary. Therefore, since imaging may help elucidate the cause, familiarity with the possible underlying conditions and their imaging features is important for abdominal radiologists.

## Biliary anatomy

The conventional biliary anatomy encompasses intrahepatic and extrahepatic bile ducts. The intrahepatic bile ducts originate from the bile canaliculi, tiny channels between hepatocytes that merge to form small ductules, known as ducts of Hering, which converge to form small (interlobular and septal) and large (area and segmental) bile ducts [3, 4].

**Fig. 1:**
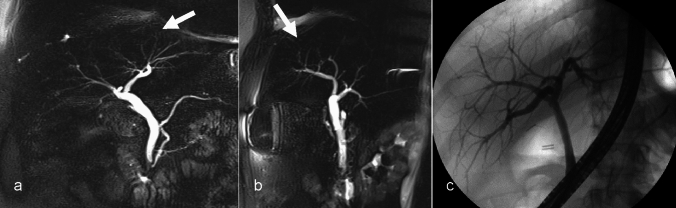
Magnetic resonance cholangiopancreatography maximum intensity projection image (arrows, **a**–**b**) demonstrates normal branching of the bile ducts with the smallest branches at the periphery with gradual increase in caliber of ducts toward the hepatic hilum. Similar findings are seen on Endoscopic retrograde cholangiopancreatography (image **c**)

The branching pattern of the intrahepatic ducts adheres to the hepatic segmental anatomy. The right anterior section duct, draining segments 5 and 8 of the liver, converges with the right posterior section duct, draining segments 6 and 7, to form the right hepatic duct. In a similar manner, the left medial section duct, which drains segments 4, and the lateral section duct, draining segments 2 and 3, combine to form the left hepatic duct. The caudate lobe tends to have variable drainage, draining into both the right and left hepatic ducts [3, 5]. The common hepatic duct (CHD) is formed by the joining of the right and left hepatic ducts at the hilum, which travels via the hepatoduodenal ligament. The cystic duct joins the CHD to form a common bile duct, which drains into the papilla of Vater in 2nd portion of the duodenum. Regardless of classic anatomy or variants in the anatomy of bile ducts, the hepatic artery, portal vein, and bile duct form a triad. The size of this triad is smallest at the periphery of the liver and gradually increases in size towards the hepatic hilum (Fig. 1) [6].

## Role of imaging and patterns of biliary injury

Magnetic resonance cholangiopancreatography (MRCP) is the initial imaging technique for evaluating biliary injury. It is preferred over Endoscopic retrograde cholangiopancreatography (ERCP) since it preserves the sterile biliary tree, is less invasive, cost-effective, and avoids potential procedural complications such as pancreatitis [7–9]. MRCP provides a comprehensive assessment of both intrahepatic and extrahepatic bile ducts. However, the patterns and severity of intrahepatic bile duct loss vary based on the severity and underlying cause of the disease. While MRCP is invaluable for non-invasive evaluation, limitations may arise in visualizing peripheral bile ducts due to factors such as patient motion, respiratory motion artifacts, presence of ascites, suboptimal imaging parameters, and susceptibility artifacts from surgical clips. The role of ERCP has evolved from primarily diagnostic to predominantly therapeutic interventions, including sampling, stone extraction, and dilation or stenting of strictures. In cases where MRCP is limited, ERCP remains crucial for directly evaluating peripheral bile ducts and addressing obstructive or pathological findings through therapeutic interventions.

In ductopenia, imaging patterns are classified by severity, from biliary strictures to progressive loss. When assessing patients with cholestasis and possible ductopenia, key goals include identifying the cause of intrahepatic cholestasis, ruling out extrahepatic obstructions, confirming ductopenia, and assessing the extent of bile duct damage. Certain diseases have distinctive imaging characteristics aiding diagnosis, while others require correlating secondary signs with clinical symptoms. In some ductopenic diseases, bile ducts can regenerate, but in VBDS, bile duct loss leads to progressive cholestasis, fibrosis, atypical ductular proliferation, and eventually cirrhosis or liver failure [1].

## Pathology and laboratory evaluation

**Fig. 2:**
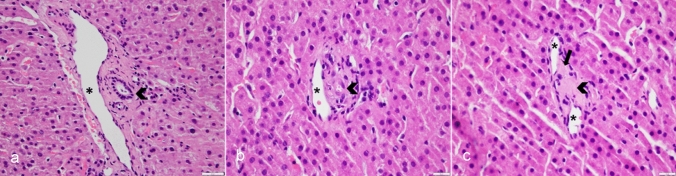
Histology pictures illustrating normal portal tract and progression to ductopenia in a patient with chronic allograft rejection. High magnification hematoxylin and eosin stain photomicrographs of portal areas and surrounding hepatocytes demonstrates spectrum of bile duct changes in portal tracts with progression to ductopenia (arrowhead, **a**: intact, normal-appearing bile duct; **b**: damaged, degenerating bile duct; **c**: absence of bile duct) and development of fibrosis (arrow, **c**). Portal vein branches are marked by asterisks (*, **a**–**c**)

Ductopenia involves progressive loss of bile ducts, but it is important to consider normal variations in the liver. Up to 7% of portal tracts may not have any visible bile ducts even in a healthy liver [10]. With regard to a standard for adequacy of a needle biopsy of the liver, most experts agree that 11 or more portal tracts within a specimen 2–3 cm long provide for an adequate assessment of portal-based pathology, although there is variable acceptance of this number and some instances fewer portal tracts can still provide for an accurate diagnosis. A 18-16-gauge caliber needle is used for non-focal liver biopsy to obtain an adequate sample [11]. In light of this, the pathologic designation of the process of bile duct loss can be nuanced, but by definition, a diagnosis of ‘ductopenia’ requires the absence of identifiable bile ducts in greater than 50% of observed portal areas in an otherwise adequate non targeted biopsy specimen (Fig. 2) [1, 2].

## Etiologies

### Primary biliary cholangitis

Primary biliary cholangitis (PBC) previously known as “primary biliary cirrhosis” is a rare autoimmune disease characterized by chronic injury to biliary epithelial cells. Both genetic and environmental factors contribute to its development. Genetically, there is increased incidence in families with a history of the disease due to shared genetic factors, and studies show high concordance in identical twins. Environmental factors, including smoking, exposure to hair dye, pollutants, and toxic waste are believed to play a role [12]. PBC predominantly affects adult women (approximately 90%), between the ages of 40–60 [13, 14]. In North America, the incidence rate is 2.75 per 100,000 population, with a female-to-male ratio of 4-10:1 [12].

PBC can be detected incidentally in asymptomatic patients through unrelated biochemical tests. Symptoms like pruritus and fatigue are common, sometimes preceding jaundice [13, 15]. Chronically elevated serum alkaline phosphatase without known cause may raise suspicion for PBC. Diagnosis relies on serum antibodies, primarily antimitochondrial antibodies (AMA), with a high sensitivity (84.5%) and specificity (97.8%). In AMA negative patients, the presence of PBC-specific antinuclear antibodies, such as sp100 and gp210, can establish diagnosis [16]. Liver biopsy is typically unnecessary unless serology is negative or other liver conditions are suspected [17].

Histologically, PBC progresses from initial focal inflammation around bile ducts to end-stage ductopenia. Lymphocytic cholangitis and the diagnostic hallmark, the 'florid duct lesion,' characterized by granulomatous inflammation around an injured bile duct, are key identifiers. As the disease progresses, fibrosis spreads from the portal areas, with bile duct loss intensifying [18]. Subsequent stages involve increasing inflammation, fibrotic distortion and ultimately cirrhosis [19].

**Fig. 3:**
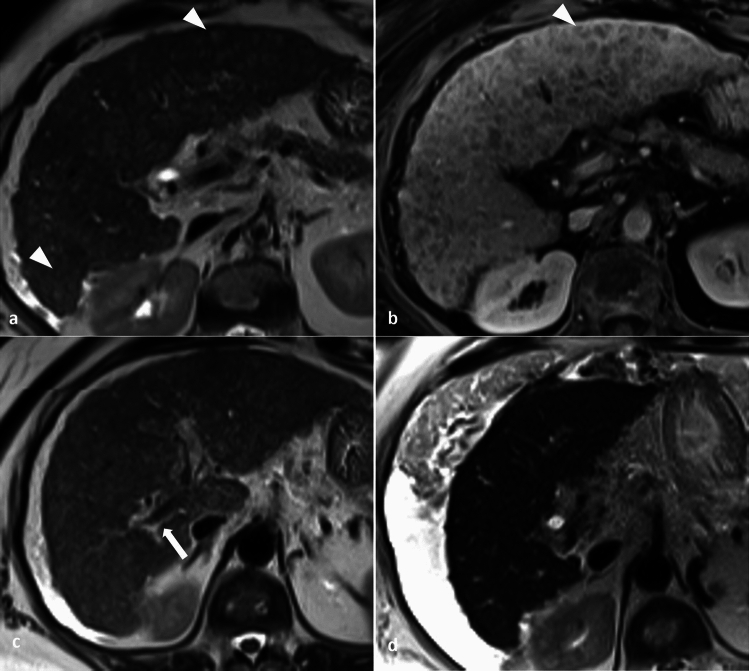
A 67-year-old female diagnosed with Primary Biliary Cholangitis at the age of 47. Axial T2 weighted MRI shows “periportal halo sign” as tiny, rounded areas of low signal intensity (arrowheads, **a**) and on postcontrast T1-weighted (arrowhead, **b**) around small peripheral portal vein branches distributed diffusely in both hemi-livers resulting in a markedly heterogeneous appearance. T2- weighted (arrow, **c**) shows periportal hyperintensity or “cuffing” around the right portal vein branch. MRI done at the age of 67 (image **d**) shows the absence of both periportal halo sign and periportal cuffing with the development of cirrhosis

In the workup for cholestasis, a right upper quadrant ultrasound is often the initial step, helping distinguish between extrahepatic causes of cholestasis, where biliary dilation is typically present, from intrahepatic causes like PBC, where biliary dilation is absent. MRI may reveal a periportal halo sign which manifests as round areas of low signal intensity on T1 and T2 weighted images around the portal venous branches with no accompanying mass effect [20]. Other imaging features, like heterogeneity on T2-weighted MRI and lacelike fibrosis on contrast-enhanced MRI, are indicative of disease progression [21–25]. Periportal cuffing due to edema is seen as periportal hyperintensity on T2WI around the large portal vein branches and is due to a combination of inflammation, increased cellularity, and biliary ductular proliferation, although it is not specific to PBC and is less common in advanced stages of PBC (Fig. 3) [21, 22, 25–27]. Cross-sectional imaging may also reveal lymphadenopathy and hepatomegaly, further aiding diagnosis [23, 24].

MRCP aids in differentiating PBC from PSC and obstructive cholestasis. Typically, PBC affects small intrahepatic bile ducts (< 100 µm), which explains the normal appearance of the biliary tract in MRCP images [19]. One study found mild irregularities in the intrahepatic bile duct in PBC affecting less than 2% of the cases [25].

Ursodeoxycholic acid (UDCA) is the first-line treatment for PBC. It slows down disease progression, improves transplant free survival, and is typically taken for life [17].

### Primary sclerosing cholangitis

Primary sclerosing cholangitis (PSC) is a chronic cholestatic disorder with multiple postulated etiologies, yet considered idiopathic. Its pathogenesis involves a combination of genetic predisposition, immune dysfunction, and environmental factors. Associations with specific HLA haplotypes highlight a genetic basis that predisposes individuals to immune responses aberrantly targeting the bile ducts [28]. These immune responses may be initiated or exacerbated by environmental triggers, such as microbial exposures, leading to chronic inflammation and fibrosis in the bile ducts, which can cause strictures, cholestasis, and eventually liver failure. Additionally, the strong association of PSC with inflammatory bowel disease, likely due to shared immune dysregulation and imbalances in gut microbiota, underscores the significant role of autoimmunity and microbial factors in its pathogenesis [28, 29]. There is a male predilection and increased incidence in the United States and Northern Europe, with a mean age of diagnosis of 40 years [30].

Patients with PSC may be asymptomatic or present with symptoms ranging from right upper quadrant pain and fever due to active cholangitis, to pruritus, jaundice, and fatigue from biliary obstruction or signs of cirrhosis such as abdominal distension from ascites if the diagnosis is delayed [29]. Diagnosis is made in the setting of chronic cholestatic laboratory abnormalities such as elevated alkaline phosphatase, gamma glutamyl transferase, moderate transaminitis plus multifocal bile duct stricturing seen on MRCP or ERCP, in the absence of another causative etiology of sclerosing cholangitis [9, 31]. PSC is therefore a diagnosis of exclusion.

PSC is often challenging to characterize histologically; thus, when diagnostic imaging (i.e., large-duct PSC) is conclusive, a biopsy is usually neither indicated nor helpful due to the liver’s patchy and variable physiologic manifestation. Biopsy findings often range from normal to nonspecific signs of extrahepatic biliary obstruction such as portal-based edema and mild ductular reaction. While concentric periductal fibrosis ('onion skin') is a classic diagnostic finding but may not always be present. Other features may include lymphocytic inflammation of the bile duct epithelium. As the disease progresses, bile ducts may atrophy or 'wither,' leading to their obliteration and loss, as hepatic fibrosis advances [32].

Imaging features of PSC include multifocal bile duct stricturing with a beaded appearance from intervening mildly dilated segments that can be intrahepatic, extrahepatic, or most commonly both. With chronicity, visualization of peripheral bile ducts is lost as they become fibrosed and the liver takes on a cirrhotic morphology with areas of confluent fibrosis. Sites of active cholangitis, hepatolithiasis, and hepatic abscesses can occur with obstruction causing upstream stasis and inflammation. Patients with PSC have an increased risk for cholangiocarcinoma, that may present as a high-grade stricture, polypoid luminal mass, or an intrahepatic focal lesion [31].

**Fig. 4:**
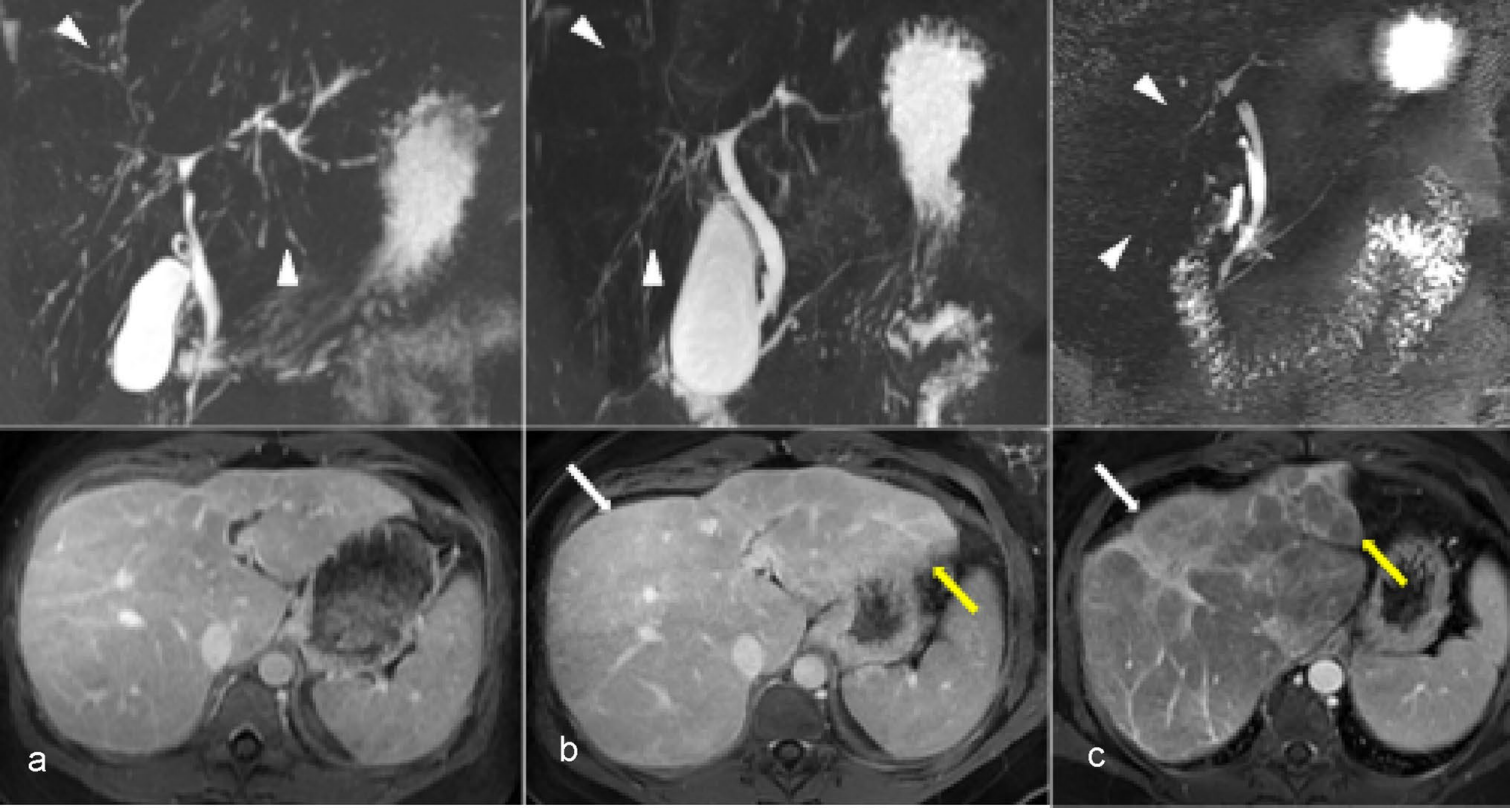
A 29-year-old female with Ulcerative colitis since the age of 14 years old presents with cholestasis. Magnetic resonance cholangiopancreatography (MRCP) maximum intensity projection and axial T1-weighted post-contrast images at the time of diagnosis (image **a**); at 4 years (image **b**) and at 12 years (image **c**) are shown. The MRCP demonstrates classic stricture-dilatation of the intrahepatic bile ducts (arrowhead, **a**). With disease progression, there is progressive obliteration of the bile ducts (arrowheads, **b**–**c**). The liver also shows progressive decrease in volume (yellow arrows, **b**–**c**), worsening of fibrosis (white arrow, **b**–**c**) and development of cirrhosis

As cholestasis from PSC progresses, obliteration of peripheral ducts leads to a “pruned” appearance of the biliary tree on cholangiography which is reflected pathologically by progressive periductal fibrosis, chronic inflammation, and ischemic atrophy of biliary epithelia results in stricturing and ductopenia (Fig. 4). Thus, PSC is a potential etiology of VBDS and biliary cirrhosis [9, 33]. The majority of adult patients with ductopenia have either primary biliary cirrhosis, PSC, or a PSC-PBC overlap syndrome. A key differentiating feature of PSC is multifocal strictures resulting in upstream multifocal dilatation of the smaller and peripheral bile ducts. Multifocal biliary dilation is often the first imaging sign, particularly if a CT is obtained first [34].

UDCA act by inducing hepatobiliary secretion, inhibiting apoptosis, and protecting cholangiocytes against the toxic effects of hydrophobic bile acids. It is used for PSC in combination with endoscopic interrogation and dilation of high-grade and relevant strictures [34]. The only curative treatment for PSC is liver transplantation.

### Ischemic cholangitis

The bile ducts are prone to ischemic injury due to their reliance on primarily arterial blood supply, in contrast to the liver, which has a dual blood supply from the portal vein and hepatic artery. The peribiliary vascular plexus is supplied by the right hepatic artery from above, the retroduodenal and retroportal arteries from below, and has a rich supply of transcapsular collaterals [35]. As a consequence, occlusion of the hepatic artery is generally has limited clinical significance except in liver transplantation, where collateral pathways are severed [36]. Outside of liver transplantation, where ischemic cholangiopathy most commonly occurs in the setting of a patent hepatic artery due to an ischemic event such as shock or cardiac arrest, toxic/ischemic injury to the small vessels of the peribiliary plexus (e.g., intraarterial chemotherapy), or vasculitis with obliteration of the peribiliary arterioles [31].

Ischemic cholangiopathy presents clinically with laboratory evidence of cholestasis and is often a leading diagnostic consideration in a critically ill patient with persistent liver chemistry abnormalities despite recovery from the original inciting event. The pathologic findings are similar to other forms of VBDS, though additional findings may include fibrous thickening, thrombosis, or obliteration of the peribiliary arterioles [1]. More specifically, the presence of bile duct epithelial cell necrosis and sloughing with biliary cast formation, when present, is the histopathologic finding most diagnostic of ischemic cholangiopathy in an appropriate clinical context.

**Fig. 5:**
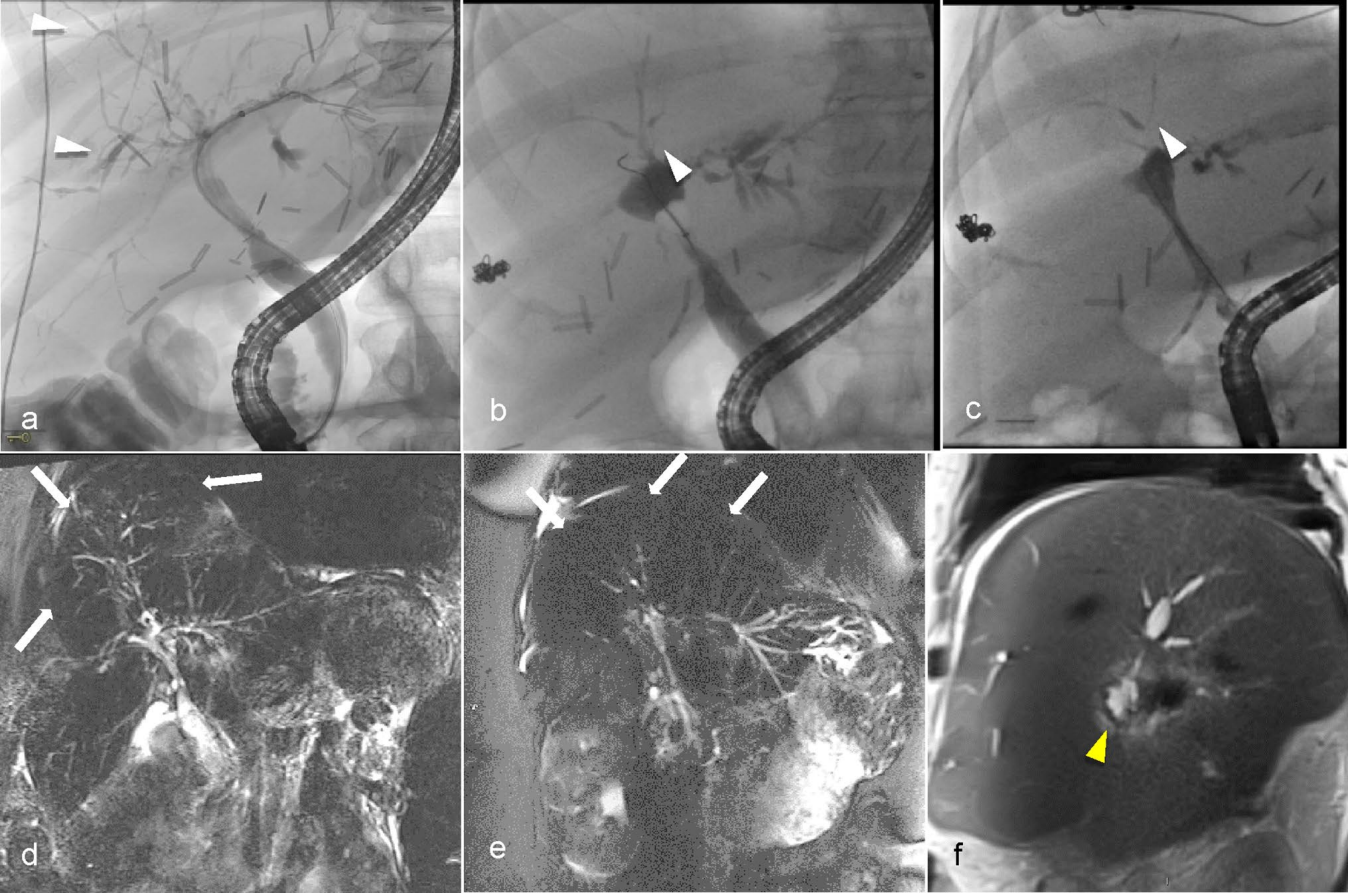
A 53-year-old female with orthotopic liver transplant approximately 7 years ago with hepatic artery stenosis and ischemia induced chronic biliary anastomotic stricture requiring internalized PTC drain and repeat biliary stenting. The hepatic artery stenosis was treated with angioplasty. The patient had persistent chronic cholestasis, elevated alkaline phosphatase and transaminase. The Endoscopic retrograde cholangiopancreatography (ERCP) images demonstrate progressive decrease in the number and caliber of bile ducts over the years. (arrowheads, **a**–**c**) due to ischemic damage to the bile ducts. ERCP at baseline (image **a**); at 6 years (image **b**) and at 7 years (image **c**) are shown. The Magnetic resonance cholangiopancreatography maximum intensity projection images demonstrate comparable findings to the ERCP, showing progressive decrease in the number and caliber of the bile ducts (arrows, **d**–**f**) and development of biloma (arrowhead, **f**) over time

Ischemic cholangiopathy after liver transplantation can manifest in four distinct patterns. The most severe, “Diffuse Necrosis”, involves widespread abnormalities and narrowing of the intrahepatic bile ducts within two months after transplant. “Multifocal Progressive” shows initial mild stenosis of the second-order and peripheral ducts that progressively worsens. The “Confluence Dominant” pattern is characterized by strictures localized at the biliary confluence, whereas the “Minor Form” presents mild early-stage abnormalities that do not progress to extensive strictures [37]. Commonly it manifests on imaging as multifocal stricturing of the intrahepatic and extrahepatic bile ducts [38]. Less commonly, it may present as biliary necrosis with the development of multiple peribiliary collections or bilomas that communicate with the biliary tree. Rarely, it may present as ductopenia in which ischemic biliary injury results in bile duct loss and VBDS [2]. As in other causes of ductopenia, the imaging findings are nonspecific, but a relative paucity of intrahepatic bile ducts may be evident on MRCP (Fig. 5). Prior imaging studies demonstrating vascular complications such as arterial thrombosis or stenosis can also be helpful in arriving at this diagnosis. Liver transplantation is frequently necessary and is considered curative [39].

### Chronic rejection following liver transplantation

Chronic rejection occurs in 3–17% of patients after liver transplantation [40]. Bile duct loss is a typical pathologic finding of chronic rejection, often attributed to a combination of T-cell mediated cytotoxic injury to the bile ducts and obliterative arteriopathy resulting in ischemic biliary injury [41].

Histologically, acute T-cell mediated allograft rejection manifests as a portal-based inflammatory infiltrates, particularly within the bile duct epithelium, causing epithelial damage. This inflammation is usually rich in lymphocytes, sometimes with eosinophils. Chronic T-cell mediated rejection shows a spectrum of changes: early signs include senescence-related bile duct epithelial changes and bile duct loss in less than 50% of portal areas. This may progress to hepatocyte necrosis extending into lobular parenchyma. More advanced chronic rejection is marked by frank ductopenia (loss of bile ducts in ≥ 50% of portal areas), loss of hepatic arterioles, fibrosis, and overt histopathologic features of cholestasis [42].

**Fig. 6:**
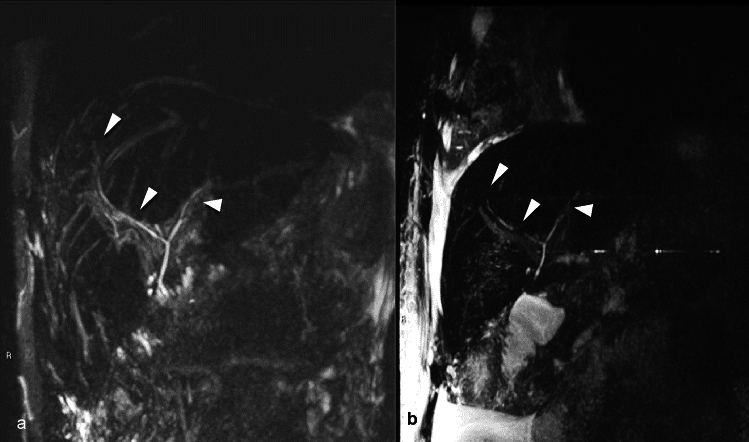
A 24-year-old male with orthotopic liver transplant for autoimmune hepatitis, after two previously failed transplants due to chronic rejection and complications from hepatic arterial thrombosis, presents with increased bilirubin levels of 30 mg/dL. MRI with Magnetic resonance cholangiopancreatography performed two months post-transplantation shows no dilation in the central bile ducts (arrowheads, **a**). The peripheral bile ducts are poorly visualized, raising concerns for ductopenia. A follow-up imaging six months later demonstrates multiple levels of bile duct obliteration (arrowheads, **b**) and non-visualization of the peripheral ducts. The persistent hyperbilirubinemia and bile duct loss, in the absence of hepatic arterial abnormalities, raised concerns for chronic rejection, which was confirmed by liver biopsy

Clinically, patients with chronic rejection or VBDS present with elevations in serum liver enzymes with a cholestatic liver injury pattern i.e., elevated alkaline phosphatase and gamma-glutamyl transferase [43]. Secondary signs such as liver fibrosis and cirrhosis in the setting of increasing jaundice and cholestatic markers in a patient with a history of acute rejection may be the only imaging clues to the correct diagnosis [44]. On MRCP, chronic rejection or ductopenia may manifest as a relative paucity of small intrahepatic bile ducts (Fig. 6) [45]. As with other forms of cholestatic liver injury, excretion of hepatobiliary contrast is often diminished or absent. In a patient with prior transplantation and laboratory evidence of cholestasis, the primary role of imaging is to exclude biliary stricturing or obstruction.

Chronic rejection may be reversible with optimized immunosuppression; however, the association of ductopenia carries a poor prognosis [46]. In patients with chronic rejection and irreversible allograft dysfunction, repeat transplantation is generally required [47].

### Drug-induced injury

Drug-induced ductopenia is an idiosyncratic reaction linked to various pharmaceuticals, including antibiotics (e.g., amoxicillin-clavulanate), antiepileptics (e.g., phenytoin), antiretrovirals, interleukins, and immune checkpoint inhibitors [48, 49]. Drug-induced injury can present as isolated cholestasis or commonly as a mixed form (cholestasis with hepatitis) [50]. Ductopenia typically manifests 1–6 months post-acute liver injury, where inflammatory responses primarily target cholangiocytes, potentially leading to bile duct degeneration and loss.

The symptoms can range from fatigue and abdominal discomfort to dark urine and pale stools. In acute cholangitis presentation, fever, shivering, and abdominal pain may precede jaundice [50]. Signs of bile duct damage are frequent in the acute hepatitis phase, while bile duct loss indicates a more chronic stage [51]. Diagnostic workup includes autoimmune markers, viral hepatitis serologies, and immunoglobulin levels to rule out alternative cholestasis causes in a patient with persistent elevations in serum alkaline phosphatase and bilirubin following onset of the drug. Drug levels and metabolites may be evaluated in specific cases [52].

Histopathologic findings in drug-induced liver injury vary widely, ranging from acute to chronic changes. Acute injury may resolve by itself or evolve into chronic phases if the offending agent continues. This typically presents as mixed portal-based inflammation with bile duct injury and degenerative changes in the biliary epithelium. While lobular parenchyma often shows mild inflammatory changes, cholestasis within hepatocytes and canaliculi is usually more pronounced than the degree of lobular or portal-based inflammation, a condition known as 'bland cholestasis'. Over time, chronicity may lead to progressive loss of intrahepatic bile ducts and fibrosis [52, 53].

**Fig. 7:**
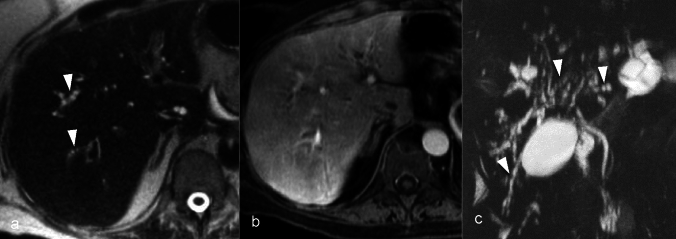
52-year-old female with metastatic clear cell RCC, treated with Ipilimumab + Nivoluman presented with acute rise in liver function test. Axial T2W MRI image demonstrates beaded appearance of the bile ducts (arrowheads, **a**). Post contrast venous phase T1W MRI shows heterogeneous enhancement of the liver parenchyma (image **b**). Magnetic resonance cholangiopancreatography maximum intensity projection shows beaded dilation of the bile ducts (arrowheads, **c**). Liver biopsy findings included acute cholestasis with bile duct injury and ductular reaction. The findings were consistent with a diagnosis of drug induced cholangiopathy after excluding other etiologies. The diagnosis was concordant with the clinical diagnosis of immunotherapy induced cholangitis

Imaging findings, though non-specific, can include bile duct dilation, irregularities resembling sclerosing cholangitis (Fig. 7), or hepatic inflammatory changes in acute phases. These manifestations may progress to more severe conditions such as ductopenia, portal tract inflammation, and fibrosis with continued drug exposure.

**Fig. 8:**
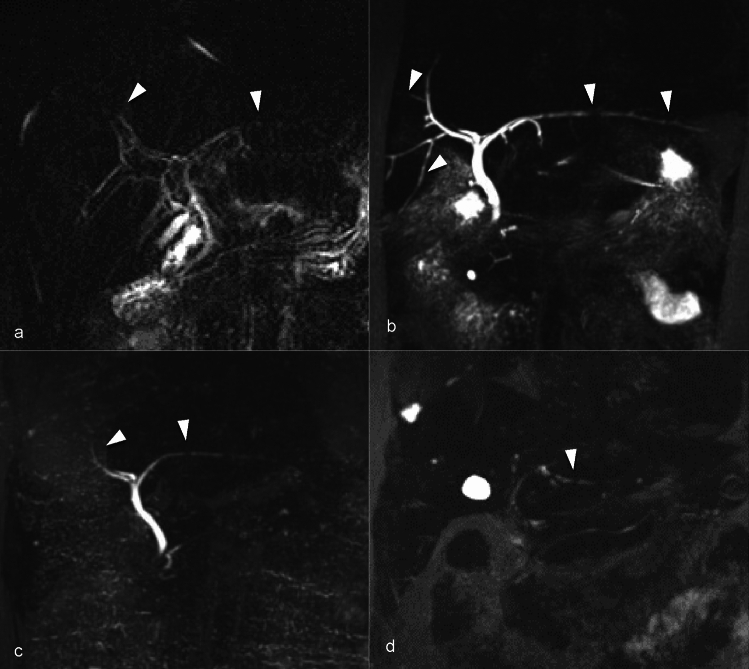
45-year-old female with cholestatic hepatitis with biopsy proven vanishing duct syndrome due to weight loss drug, Zantrex 3. The initial Magnetic resonance cholangiopancreatography (MRCP) imaging shows non-dilated bile ducts with paucity of peripheral duct (arrowheads, **a**). Improvement in the cholestatic liver enzyme pattern and symptoms were observed on two months follow-up, following withdrawal of the drug. The MRCP maximum intensity projection image shows better delineation of the peripheral ducts (arrowheads, **b**) due to ductal regeneration. MRCP maximum intensity projection image at three years follow-up shows worsening of the disease, with progressive obliteration of central and peripheral ducts due to reuse of the drug (arrowheads, **c**–**d**)

MRI with MRCP is supportive in diagnosis, with MRCP being particularly useful for detecting intrahepatic bile duct irregularities or dilation and for sequential monitoring of drug-induced injury progression or treatment response over time, although these may not be apparent in early disease stages (Fig. 8). Additionally, MRI provides information regarding any associated liver damage or parenchymal changes including periportal edema and heterogeneous parenchymal enhancement during arterial phase [54, 55].

Treatment prioritizes resolving the underlying cause and withdrawing the implicated drug. UDCA may be beneficial for cholestasis. Corticosteroids are considered for immune-mediated cases, with plasmapheresis as an option in refractory situations. In cases of progressive liver failure, liver transplantation remains the definitive treatment.

### Infection

Viral and bacterial cholangitis are inflammatory conditions of the bile duct system, caused by different pathogens. Bacterial or viral infectious cholangitis very uncommonly progresses to ductopenia. However, persistently elevated abnormal laboratory results should indicate the possibility of developing ductopenia. CMV is the most common virus associated with ductopenia occurring in neonates or immunocompromised hosts [48, 56].

Viral cholangitis is often a result of hepatitis viruses, leading to symptoms like jaundice, abdominal pain, and fever. Whereas, bacterial cholangitis is typically caused by bacteria ascending from the intestine, often associated with bile duct obstructions such as gallstones. Its symptoms include severe abdominal pain, jaundice, fever, and chills, and it may lead to sepsis if untreated. Diagnosis relies on clinical symptoms, elevated liver enzymes, and infection markers.

Histopathologically, sepsis in the liver often presents with cholestasis, visualized as inspissated bile in dilated ductules, termed 'cholangitis lenta,' often accompanied by neutrophilic inflammation and microabscess formation. Additionally, liver biopsies may reveal ischemic damage like centrilobular necrosis and microvesicular fatty change. Infectious cholangitis damages the biliary epithelium, leading to bile duct obliteration and loss, attributed to bacterial toxins and immune-mediated responses from bacterial and viral infections. This process may culminate in duct replacement with fibrous, elastic fiber-rich lesions, similar to those seen in PSC. While diagnosis often depends on serology, specific pathogens like CMV manifest distinct histopathological features, including 'owl’s eye' inclusions and a viral cytopathic effect in hepatocytes, vascular endothelium, and biliary epithelium, potentially progressing to bile duct destruction [1, 57].

**Fig. 9:**
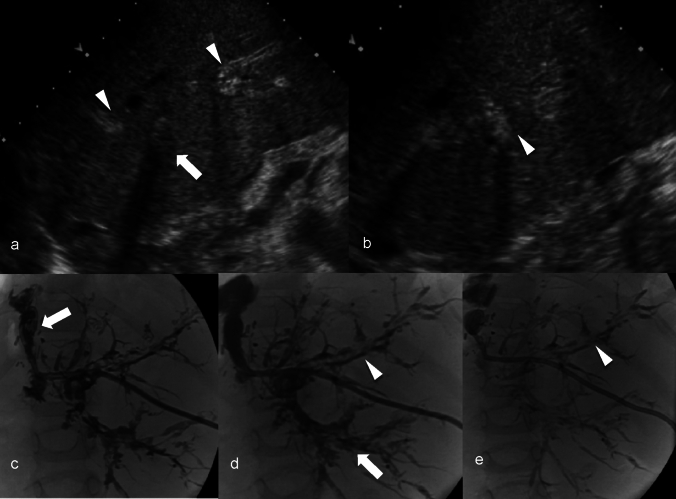
Ultrasound images demonstrate multiple echogenic foci scattered in the liver within the portal triads (arrowhead, **a**–**b**) related to inflammation around the biliary ducts with a few bile ducts demonstrating posterior acoustic shadowing due to ductal stones (arrow, **a**–**b**) in a patient with history of recurrent cholangitis, fevers, and massively elevated alkaline phosphatase. Endoscopic retrograde cholangiopancreatography images demonstrate diffusely irregular and dilated ducts (arrow, **c**–**d**) with filling defects (arrowhead, **d**–**e**)

The imaging findings are nonspecific; however, secondary signs related to viral hepatitis and cholangitis may be helpful in the correct clinical setting. MRI shows differential perfusion of the liver parenchyma adjacent to the bile ducts in cases with bacterial cholangitis. The bile ducts may show wall thickening and contrast enhancement in the acute phase. Repeated episodes of cholangitis result in biliary strictures, stasis, and hepatolithiasis (Fig. 9). The biliary stricture in recurrent pyogenic cholangitis shows an arrowhead appearance where the peripheral bile ducts show decreased branching and abrupt tapering of the peripheral ducts and disproportionate central and extrahepatic bile duct dilation. The progression of infection can culminate in complications such as hepatic abscess, biloma formation, and portal vein thrombosis [58, 59].

Treatment in viral cholangitis focuses on antiviral medications to combat the underlying viral infection and supportive care to manage symptoms and complications. Treatment for bacterial cholangitis consists of antibiotics with endoscopic or percutaneous biliary drainage to stone removal and ductoplasty [58]. Both conditions require prompt medical attention to prevent serious complications and ensure effective management.

.

### Sarcoidosis

Sarcoidosis is a multisystem granulomatous disease that can involve the liver and bile ducts. The exact etiology of sarcoidosis remains unknown; however, it is believed to involve a combination of genetic predisposition and environmental triggers that lead to an abnormal immune response. It commonly presents in adults between the ages of 20 and 40 years and is often more severe in African Americans. In the United States, sarcoidosis appears to be more common in females than in males [60]. Biliary sarcoidosis is characterized by the formation of non-caseating granulomas within the liver and bile ducts, leading to ductopenia or loss of bile ducts in severe cases.

Most patients with hepatic sarcoidosis are asymptomatic, though biliary sarcoidosis can present with jaundice due to either mass effect on the bile ducts by the enlarged porta hepatis lymph nodes or ductal involvement. Laboratory evaluation reveals elevated alkaline phosphatase and gamma-glutamyl transferase, with or without elevated bilirubin levels. Angiotensin-converting enzyme levels may be elevated in sarcoidosis, but this is not specific to the disease. For definitive diagnosis, liver function tests, serological markers for other liver diseases, and immunological tests to rule out alternative causes are essential [61].

Histopathology remains the cornerstone for diagnosis, demonstrating non-caseating granulomas in the liver and bile ducts. Over time, chronic granulomatous inflammation can lead to scarring and fibrosis around the bile ducts, resulting in significant reduction or disappearance of bile ducts within affected areas of the liver. This can lead to cholestasis, as the reduction in bile ducts impairs bile flow from the liver to the intestine. In advanced cases, the ongoing inflammation and granuloma formation can lead to more widespread fibrosis throughout the liver, potentially progressing to cirrhosis [62].

**Fig. 10:**
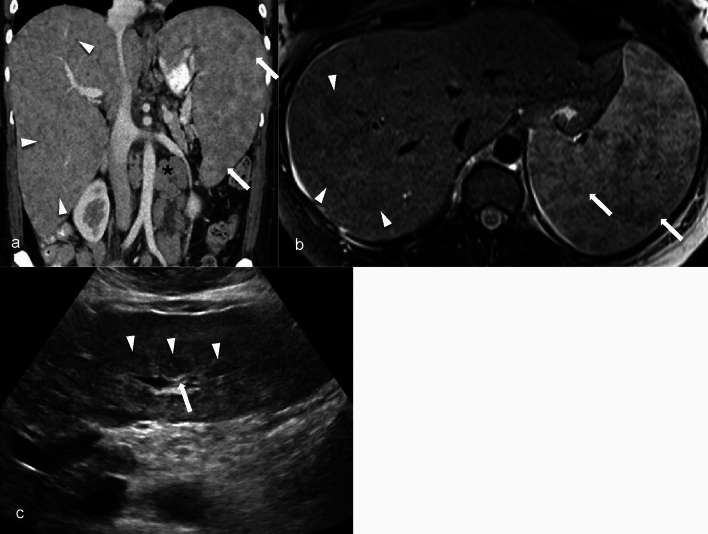
39-year-old female with a history of sarcoidosis and cholestatic hepatitis. Coronal contrast enhanced CT demonstrates enlarged liver and spleen with heterogeneous appearing parenchyma secondary to multiple hypodense nodules scattered throughout the liver (arrowheads, **a**–**b**) and spleen (arrows, **a**–**b**). Multiple enlarged retroperitoneal lymph nodes are present (asterix, **a**). Axial T2W MR image shows hypointense nodules scattered throughout the liver (arrowheads, **b**) and spleen parenchyma (arrows, **b**). Ultrasound images demonstrate multiple hypoechoic nodules in the liver (arrowheads, **c**) with mass effect on the portal triads (arrow, **c**) from non-caseating granulomas

Imaging findings in biliary sarcoidosis can vary and may be subtle, especially in asymptomatic patients who are undiagnosed at the initial presentation. Common imaging features include hepatomegaly, splenomegaly, and multiple nodular lesions in the liver and spleen which can become confluent and exert mass effect on the bile ducts and lymphadenopathy (Fig. 10).

**Fig. 11:**
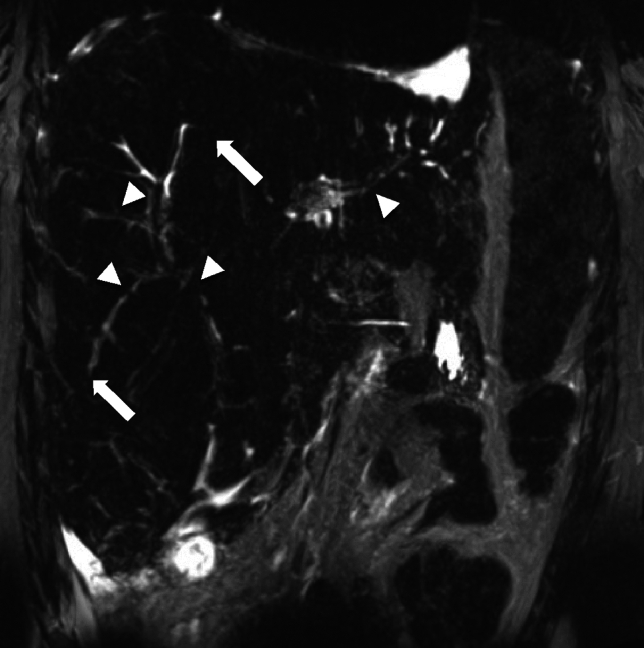
Similar patient as in Fig. 10, 39-year-old female with a history of sarcoidosis and cholestatic hepatitis. The Magnetic resonance cholangiopancreatography maximum intensity projection image demonstrates non-dilated bile ducts with scattered areas of narrowing (arrowheads) and obliteration of the peripheral duct (arrow) from biliary involvement in sarcoidosis

With biliary involvement, biliary obstruction due to mass effect from enlarged porta hepatis lymph nodes or bile duct irregularities is present. The range of imaging findings depends on the severity of the disease at the time of diagnosis. As the disease progresses, obliteration of bile ducts resulting in ductopenia, findings of fibrosis and cirrhosis similar to other etiologies are seen (Fig. 11) [62].

Management of biliary sarcoidosis and ductopenia focuses on controlling the granulomatous inflammation and managing symptoms of cholestasis. Corticosteroids are the mainstay of treatment to reduce inflammation. In cases of refractory disease or corticosteroid intolerance, immunosuppressive agents such as methotrexate or azathioprine may be used. Ursodeoxycholic acid can help alleviate cholestatic symptoms [63].

### Hodgkin lymphoma

Hodgkin lymphoma (HL) is an uncommon cause of ductopenia, observed only in a small subset of HL patients. The connection between the two conditions was first reported by Hubscher and colleagues in 1993 when three patients with HL presented with severe intrahepatic cholestasis [64]. HL originates in the lymphatic system and is characterized by the presence of abnormal Reed-Sternberg cells in the lymph nodes and other lymphatic tissues. The mechanism of bile duct injury in HL-induced VBDS is believed to be a paraneoplastic phenomenon. The cytotoxic cytokines released by HL cells directly or through T cell-mediated mechanisms lead to autoimmune destruction of the biliary epithelial cells, causing VBDS [65, 66].

In some patients, an association between HL and VBDS has been proposed, along with PSC and inflammatory bowel diseases. This association may be attributed to certain host genetic factors. Some patients with HL-related VBDS have been found to have defects in the MST1 gene locus or molecular abnormalities in genes involved in bile acid transport or synthesis [67–69]. Jaundice and pruritus were reported in all patients with HL-induced VBDS in a literature review conducted by Wong et al. Other common presenting features included weight loss, night sweats, fever, neck lymphadenopathy, enlarged liver, and spleen [68].

The imaging characteristics of Hodgkin's lymphoma-induced ductopenia usually appear normal in the early stages or may exhibit either diffuse or localized narrowing and irregularity of intrahepatic and extrahepatic bile ducts without significant dilatation. The bile ducts may also exhibit wall thickening or a beaded appearance, characterized by alternating areas of narrowing and dilation similar to PSC. These specific features are crucial in suggesting lymphoma as a potential diagnosis in patients who present with cholestasis, particularly when obstructive causes and other common causes of ductopenia are excluded. Blurring of fat surrounding the portal vein was noted in a case [70]. Other imaging findings of Hodgkin's lymphoma can include lymphadenopathy, an enlarged liver, and spleen [70, 71].

Treating HL-induced VBDS can be challenging, mainly due to underlying liver disease. Various treatment approaches have been attempted with varying degrees of success, including using UDCA, prednisone, chemotherapy, and radiation therapy to achieve remission of HL [66, 67, 72].

### Graft-versus-host disease

Graft-versus-host Disease (GVHD) is a complication of allogeneic hematopoietic cell transplantation. The donor organ contains T-lymphocytes that are activated by cytokine release from tissue damage initiated by conditioning regimens and by bacterial polysaccharides translocated across the gut. The presence of these cytokines and the immunologically dissimilar recipient organ tissue creates a milieu that leads to the proliferation of T-cells and activation of an immunological cascade that results in tissue damage. Chronic GVHD was originally defined as a disease present at or continuing 100 days after transplant, with acute GVHD occurring before that [73]. However, it is now understood that both can occur contemporaneously. This arbitrary temporal distinction has been abandoned in favor of identifying a constellation of symptoms that identify acute and chronic GVHD [73]. Unlike other organs (skin, gut, etc.), no clinical manifestations related to the liver differentiate acute from chronic GVHD. The biliary ductal loss leading to ductopenia that characterizes VBDS is seen in chronic GVHD [2]. In general, there is no clear distinction in pathology between acute and chronic GVHD. Bile duct involvement is an early and characteristic feature of both types of GVHD. Small bile ducts and ductules involve lymphocytic infiltration and display reactive nuclear and cytoplasmic changes. As the process prolongs, periductal fibrosis and bile duct loss occur [74].

The diagnosis of acute or chronic GVHD is often based on diagnostic or distinctive features in other organs, such as liver biopsy, although necessary for documenting GVHD in the liver, has risks in the acute setting. Both acute and chronic GVHD present with a cholestatic pattern. There are no biochemical tests that differentiate the two conditions.

There are no distinctive imaging findings in GVHD. MRCP findings include normal appearing bile ducts in the initial stage to disappearance of the peripheral bile ducts in a longstanding GVHD due to continued ductal damage [75]. Transient dilation of the biliary tract has been reported but is a non-specific finding [76].

## Summary

Ductopenia and Vanishing Bile Duct Syndrome represent a continuum of biliary pathology characterized by progressive loss of bile ducts. The mechanism begins with injury to the biliary epithelium. Depending on the duration and severity of the injury, this can either lead to regeneration or, more critically, to the progressive loss of bile ducts. This loss is evident in imaging techniques, MRCP, and ERCP, which show a notable paucity of bile ducts. Clinically, this condition manifests as a persistent cholestatic pattern characterized by symptoms such as jaundice, itching, and abnormal liver function tests, particularly elevated levels of serum alkaline phosphatase. Ursodeoxycholic acid helps in providing symptomatic relief from the clinical manifestation of cholestasis. The initial response in managing this condition is to identify and address the underlying etiology, which can potentially reverse the process. However, if the damage to the biliary epithelium continues unabated, the condition progresses beyond ductopenia to Vanishing Bile Duct Syndrome, fibrosis and, eventually, to cirrhosis. At this advanced stage, liver transplantation is the definitive treatment. The key role of imaging is to rule out obstructive causes for cholestasis, followed by evaluation for specific imaging patterns or secondary findings to identify the cause and identify the paucity of the bile ducts on MRCP (Table 1).

**Table 1:** Summary of etiologies with typical imaging and pathologic findings*

Etiologies	Imaging findings	Pathology findings
Primary Biliary Cholangitis	Periportal halo sign, lacelike hepatic fibrosis	Lymphoplasmacytic infiltration, granulomatous inflammation, portal granulomas, fibrosis with bile duct loss
Primary sclerosing cholangitis	Multifocal bile duct stricturing and pruned appearance	Loss of peripheral bile ducts, concentric periductal fibrosis (onion skin), and lymphocytic inflammation
Ischemic Cholangitis	Multifocal stricturing, biliary necrosis and biloma	Cholangiocytes necrosis, sloughing with biliary cast formation
Chronic rejection	Fibrosis, cirrhosis with paucity of bile ducts	Lymphocyte-rich inflammation, loss of portal tract arterioles
Drug induced bile duct injury	Bile duct dilation, ductal irregularities, periportal edema	Mixed portal-based inflammation, degenerative changes of cholangiocytes
Infection	Bile duct wall thickening, biliary strictures, hepatolithiasis	Neutrophilic inflammation, macrophage hyperplasia, centrilobular necrosis, microvesicular fatty change, Owl’s eye inclusions (CMV)
Sarcoidosis	Bile duct irregularities, numerous nodular lesions in the liver and spleen, and lymphadenopathy	Non-caseating granulomas in the liver and bile ducts
Hodgkin’s Lymphoma	No distinctive imaging findings	Reed-Sternberg cells in liver and bile ducts
Graft versus host disease	No distinctive imaging findings	Lymphocytic infiltration, reactive nuclear and cytoplasmic changes

*Specificity and sensitivity of these imaging findings can vary, and they should be interpreted in the context of the clinical picture and additional diagnostic tests

## Data Availability

Not applicable.
